# Editorial: Roles of Ion Channels in Immune Cells

**DOI:** 10.3389/fimmu.2016.00048

**Published:** 2016-02-15

**Authors:** Leanne Stokes, Amanda B. MacKenzie, Ronald Sluyter

**Affiliations:** ^1^Health Innovations Research Institute, RMIT University, Melbourne, VIC, Australia; ^2^School of Pharmacy, University of East Anglia, Norwich, UK; ^3^Department of Pharmacy and Pharmacology, University of Bath, Bath, UK; ^4^School of Biological Sciences, University of Wollongong, Wollongong, NSW, Australia; ^5^Centre for Medical and Molecular Bioscience, University of Wollongong, Wollongong, NSW, Australia; ^6^Illawarra Health and Medical Research Institute, Wollongong, NSW, Australia

**Keywords:** ion channels, immune cells, microglial cells, lymphocytes, calcium, signalling pathways

**The Editorial on the Research Topic**

**Roles of Ion Channels in Immune Cells**

Ion channels are critical membrane proteins controlling a wide variety of cellular signaling events in many different cell types. In classical excitable cells, voltage-sensitive ion channels have well-described roles in the regulation of action potentials and contraction events. In non-excitable cells such as circulating or tissue-resident cells of the immune system, ion channels have lesser described roles in controlling cell biology. However, through enhanced pharmacological tools and gene knockouts, we are now beginning to appreciate the plethora of cellular events in immune cells that are controlled by a variety of ion channels (1). The goal of this research topic was to highlight some of the recent advances in understanding which ion channels are important and are under investigation for novel therapeutics. This collection of six articles covers microglia, myeloid cells, and lymphocytes and provides updates and insights into the roles of various channels in these cells.

Mortadza and colleagues review the latest evidence supporting a functional role for transient receptor potential melastatin (TRPM) 2 ion channels in the immune system (Syed Mortadza et al.). TRPM2 ion channels are activated by increases in reactive oxygen species (ROS) leading to Ca^2+^ signals in a range of immune cells. The authors discuss the use of TRPM2 knockout mice revealing an important role in the immune system and inflammatory diseases. Based on these studies in mouse models, the authors highlight a negative role for TRPM2 in colitis, ischemia-/reperfusion-mediated tissue damage, and neuropathic and inflammatory pain. They also discuss the importance of TRPM2 in inflammatory signaling including NLRP3 inflammasome activation leading to inflammatory cytokine production including bioactive interleukin (IL)-1β and chemokine-dependent dendritic cell function. Overall, the review demonstrates the importance of TRPM2 as a future therapeutic target for the treatment of inflammatory disease.

Stebbing and colleagues provide a mini-review of numerous ion channels expressed in microglia (Stebbing et al.). Ligand-gated channels belonging to P2X and *N*-methyl-d-aspartate (NMDA) receptor families respond to the neurotransmitters adenosine triphosphate (ATP) and glutamate, respectively. These are involved in the sensing of local microenvironments within the central nervous system and can affect microglial activation states through modulation of intracellular Ca^2+^ levels. In addition, K^+^ channels are also implicated in microglial responses including proliferation, nitric oxide generation, and cytokine secretion and, as such, are potential therapeutic targets for increasing neuroprotection in neurodegenerative disorders such as Alzheimer’s disease.

Through an original research article, Ferreira and colleagues describe the regulation of the intermediate conductance Ca^2+^-activated K^+^ channel, KCa3.1, by cyclic guanosine monophosphate (cGMP)-dependent protein kinase (PKG) via a pathway involving ROS and Ca^2+^/calmodulin-dependent protein kinase II (CaMKII) in microglia (Ferreira et al.) KCa3.1 is widely expressed in immune cells, but knowledge of KCa3.1 in microglia is hitherto limited. Through pharmacological approaches, and the use of the rat MLS-9 microglial cell line and alternative-activated primary rat microglia, the authors demonstrate that elevation in cGMP activates PKG to promote mitochondrial ROS formation. This in turn stimulates endoplasmic reticulum Ca^2+^ release, which subsequently binds to calmodulin to both open the KCa3.1 channel and activate CaMKII to increase KCa3.1 activity through a second, unknown mechanism. The authors propose that this process may serve as a positive feedback mechanism to promote KCa3.1-dependent responses in microglia under conditions such as oxidative stress.

Moving to the adaptive immune system, Davenport and colleagues have reviewed the current understanding of the Ca_V_1 subfamily of L-type voltage-gated Ca^2+^-channels expressed by lymphocytes (Davenport et al.). The authors discuss the evidence supporting the expression of Ca_V_1 ion channels in immune cells including a rare genetic disease (Timothy Syndrome) that suggests a role for Ca_V_1.2 in human immune responses. Evidence for Ca_V_1 function in B lymphocytes is largely supported by pharmacological studies, and the authors present further evidence for the expression of Ca_V_1 ion channels in B lymphocytes using a Ca_V_1.3 deficient chicken DT40 B cell line. It is concluded that Ca_V_1 channels play an important role in the immune system; however, there are many unanswered questions particularly surrounding the potential activation mechanism of immune Ca_V_1 channels.

Nohara and colleagues review the current understanding of the complex orchestration of Ca^2+^ responses in T lymphocytes and recent advances in understanding store-operated calcium channels (STIM/Orai), TRP channels, NMDA receptors, P2X receptors, and Ca_V_ channels in T lymphocytes (Nohara et al.). The authors focus on Ca_V_ channels for which there is growing evidence particularly from the recent use of Ca_V_1 channel subunit knockout mice where significant effects are observed on T lymphocyte responses. The potential interplay between CaV and STIM proteins in T cells is discussed, and the complexity surrounding regulation of intracellular (Ca^2+^) in these cells is highlighted. The authors conclude by identifying that superior immunomodulators may be developed targeting lymphocyte-specific variants of ion channels and these may be highly beneficial in future treatments for autoimmune diseases or immunosuppression required for transplants.

Finally, Rissiek and colleagues review the P2X7 receptor, an ATP-gated cation channel, in murine T lymphocytes (Rissiek et al.). The authors highlight that both extracellular ATP and nicotinamide adenine dinucleotide (NAD) can activate P2X7 on murine T lymphocytes. In contrast to ATP, P2X7 activation by NAD requires covalent adenosine triphosphate (ADP)-ribosylation catalyzed by the ecto-ADP-ribosyltransferase ARTC2.2. Notably, NAD activates only the splice isoform P2X7k and the main P2X7 subtype within murine T lymphocytes. The authors describe the roles of P2X7 in murine (and human) T cells including IL-2 production, shedding of cell surface receptors, and cell death. Lastly, the authors highlight that ATP and NAD is released during T cell isolation, which results in the preferential loss of T regulatory and natural killer T cells due to high P2X7 expression on these cells, and outline ways in which to circumvent this to improve the quality of *ex vivo* T cell preparations.

Collectively, this *Frontiers in Immunology* Research Topic provides a sample of the diversity and importance of ion channels in immune cells. We are grateful to each author and trust that this Research Topic will serve as a prompt to continue future investigations of ion channels in immune cells.

## Author Contributions

LS, AM, and RS contributed equally to this editorial article.

## Conflict of Interest Statement

The authors declare that the research was conducted in the absence of any commercial or financial relationships that could be construed as a potential conflict of interest.
